# Three-Dimensional Carbon Monolith Coated by Nano-TiO_2_ for Anode Enhancement in Microbial Fuel Cells

**DOI:** 10.3390/ijerph20043437

**Published:** 2023-02-15

**Authors:** Fan Zhao, Yini Chen, Shiyang Zhang, Meng Li, Xinhua Tang

**Affiliations:** School of Civil Engineering and Architecture, Wuhan University of Technology, Wuhan 430062, China

**Keywords:** microbial fuel cells, anode, carbon monolith, nano-TiO_2_, *Acinetobacter*

## Abstract

A three-dimensional (3D) anode is essential for high-performance microbial fuel cells (MFCs). In this study, 3D porous carbon monoliths from a wax gourd (WGCM) were obtained by freeze-drying and carbonization. Nano-TiO_2_ was further coated onto the surface of WGCM to obtain a nano-TiO_2_/WGCM anode. The WGCM anode enhanced the maximum power density of MFCs by 167.9% compared with the carbon felt anode, while nano-TiO_2_/WGCM anode additionally increased the value by 45.8% to achieve 1396.2 mW/m^2^. WGCM enhancement was due to the 3D porous structure, the good conductivity and the surface hydrophilicity, which enhanced electroactive biofilm formation and anodic electron transfer. In addition, nano-TiO_2_ modification enhanced the enrichment of *Acinetobacter*, an electricigen, by 31.0% on the anode to further improve the power production. The results demonstrated that the nano-TiO_2_/WGCM was an effective anode for power enhancement in MFCs.

## 1. Introduction

Microbial fuel cells (MFCs) are an environmentally friendly biotechnology that uses electroactive bacteria (EAB) as anode catalysts to degrade organic matter in water and directly convert chemical energy into electrical energy, which has been widely studied in electricity production, wastewater treatment, biosensors, chemical synthesis, bioremediation, etc. [1,2,3,4,5]. Power production of MFCs has increased significantly in the past decade. However, it is still at a low level which is one of the bottlenecks for practical applications of this technology. Therefore, how to improve its power density has become a big challenge for the development of MFCs. The electricity generation in MFCs is affected by many factors such as electrode material, reactor configuration, separator material, microbial community structure and operating conditions [6]. Among them, the anode material is one of the most critical factors [7,8]. The anode is the adhesion site of the EAB, the acceptor of electrons, which determines the power generation efficiency of MFCs [8]. Generally speaking, excellent anode materials should meet the requirements of good biocompatibility, large surface area, excellent conductivity and high electrocatalytic activity.

Many carbon materials (carbon cloth, carbon felt (CF) and graphite rod) are used as the anode of MFCs. However, traditional carbon anodes have inherent defects such as poor hydrophilicity, low electrocatalytic activity and low specific surface area [9]. Therefore, the modification of carbon-based anode materials has been extensively studied [10,11,12]. In addition, using natural resources to develop electrode materials with 3D porous structures, high electrocatalytic activity and enhanced hydrophilicity has become a green and efficient method to improve MFC performance [13,14,15]. The microporous structure of biochar materials is conducive to microbial colonization. Moreover, the pyrolysis of biochar materials forms carboxyl and hydroxyl functional groups on the surface of the materials, which improves the hydrophilicity. Good hydrophilicity enhances the interaction between the material interface and the electrochemically active biofilm, which can further improve microbial adhesion and extracellular electron transfer (EET) efficiency [16]. Moreover, the use of nanostructured materials to modify the electrode surface can further increase the specific surface area of the electrode and obtain excellent pseudo capacitance, which contributes to electrode reactions and long-distance EET [17,18]. The efficiency of EET is related to direct electron transfer (DET), and the efficiency of DET is related to the interaction between the electrode surface and bacterial outer membrane redox protein. However, the redox center of the enzyme is usually buried deeply in the protein, which makes it difficult for redox proteins to transfer electrons directly to the bare electrode. Therefore, a medium could be used to boost the communication between the electrode and the redox center of the protein. By coupling with EAB membrane proteins, TiO_2_ nanoparticles could act as a mediator to enhance the electron transfer from the EAB to the electrode [19]. In addition, TiO_2_ has excellent electrical properties due to its high dielectric constant and semiconductor properties, as well as favorable biocompatibility, so it can be used as an anode to improve the electrical performance of MFCs [15,20]. Therefore, 3D porous carbon coated with TiO_2_ as an anode is expected to improve the power generation of MFCs. Wax gourd (WG), cultivated throughout China, is a simple and readily available raw material for preparing biochar. It has high water content and rich fiber structure, which leads to structural collapse during natural drying. Therefore, the freeze-drying method is adopted to obtain 3D porous monolithic materials with intact fibers [21].

In this work, nano-TiO_2_/WGCM was obtained by coating TiO_2_ on a wax gourd carbon monolith (WGCM). The nano-TiO_2_/WGCM were characterized and used as high-performance anodes for MFCs. The anode bioelectrochemical performance and biocompatibility were evaluated. The composition and abundance of microbial communities on the anodes surface were also studied.

## 2. Materials and Methods

### 2.1. Preparation of the Anodes

**Figure 1:**
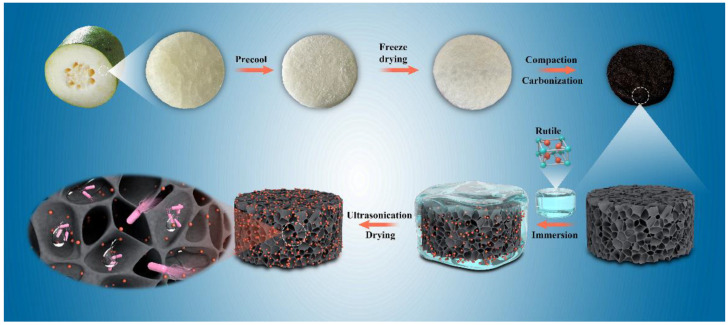
Preparation scheme of WGCM and nano-TiO_2_/WGCM.

The WGCM electrode was prepared using a simple carbonization process, as described previously [21]. The specific preparation process of nano-TiO_2_/WGCM is depicted in Figure 1.

First, the outer skin of WG was removed and the fleshy parts containing coarse fibers and sufficient moisture were cut into cylinders with a size of Φ 40 mm × 10 mm. The obtained blocks were ultrasonically cleaned with deionized water for 15 min and then placed in an ultra-low temperature refrigerator at −80 °C. Next, the blocks were placed in a freeze-drying oven and freeze-dried at −50 °C for 36 h to ensure that the frozen water in the WG tissue was completely sublimated. Finally, the blocks were carbonized in a tube furnace at 800 °C for three hours under a nitrogen atmosphere to obtain the WGCM.

A total of 2 mL PTFE and 5 mg TiO_2_ (99.8% purity, Shanghai Aladdin Biochemical Technology, Wuhan, China) were uniformly dispersed in 5 mL isopropanol under ultrasonic treatment to obtain TiO_2_ dispersion. Next, the WGCM was soaked and ultrasonically treated for 15 min in the TiO_2_ solution. Lastly, the WGCM with well-dispersed nano-TiO_2_ was removed and dried at 70 °C to acquire the nano-TiO_2_/WGCM.

### 2.2. MFC Setup and Operation

Rectangle dual-chamber MFCs with an anode chamber volume of 25 mL and a cathode chamber volume of 50 mL were constructed as previously reported [22]. WGCM, nano-TiO_2_/WGCM and CF were each used as anodes (Φ 25 × 3 mm), while Pt/C-coated CF (0.5 mg Pt/cm^2^) was employed as the cathode (Φ 25 mm × 3 mm). The anode and cathode were connected by titanium wire over a resistor of 1000 Ω. The two chambers were separated by a cation exchange membrane (CEM, Φ 40 mm, CMI-7000).

The three MFCs were inoculated with a bacterial suspension from another acetate-fueled MFC, which was started up with activated sludge from a sewage treatment plant and had been operating in the laboratory for about two years. Anolyte and catholyte were prepared as previously reported [23]. The anolyte had been sparged with nitrogen gas for 30 min to remove the dissolved oxygen and the catholyte was purged with sterile air to provide oxygen. The MFCs were studied at 25 °C in fed batch mode and the anolyte was replaced once the voltage dropped below 50 mV. Cell voltages over the external resistor was detected and stored by a data recorder system every 60 s. MFCs were considered to be stable when the maximum voltage was reproducible in three consecutive cycles.

### 2.3. Characterizations and Analysis

A scanning electron microscope (SEM, JSM-IT300, JEOL, Tokyo, Japan) was employed to observe the surface morphology. An InVia Raman microscope (InVia Renishaw, London, UK) was applied to measure the Raman spectra of WGCM and CF under the excitation of a 633 nm laser. Fourier-transform infrared spectroscopy (FTIR, Nicolet6700, Thermo Fisher Scientific, Los Angeles, CA, USA) was employed to record infrared spectra in the mid-infrared range (400–4000 cm^−1^). X-ray diffraction (XRD, D8 Advance, Bruker, Frankfurt, Germany) was used to determine the crystalline phases of WGCM and nano-TiO_2_/WGCM. X-ray photoelectron spectroscopy (XPS, ESCALAB 250Xi, Thermo Fisher Scientific, Los Angeles, CA, USA) was employed for the quantitative analysis of elements and functional groups. A Contact Angle Meter (CA, OCA35, Dataphysics, Munich, Germany) was applied to study the hydrophilicity.

The electrochemical activity of the anodic biofilm was characterized by cyclic voltammetry (CV) with an electrochemical workstation (CHI660D, Chenhua, Shanghai, China). The anode served as the working electrode, an Ag/AgCl electrode inserted in the anode chamber was the reference electrode, and the cathode was the counter electrode. The CV tests were carried out with a scanning range from −0.8 V to 0.2 V (vs. Ag/AgCl) at a scan rate of 10 or 50 mV/s. The power density curve and polarization curve were drawn from the voltage data obtained by switching the external resistor from 2000 Ω to 50 Ω.

The density of bacteria on the three anodes was measured by hemocytometry counting [24]. The microbial community structure and abundance were analyzed by Illumina Hiseq sequencing of 16S rRNA.

## 3. Results

### 3.1. Characteristics of Anodes

**Figure 2:**
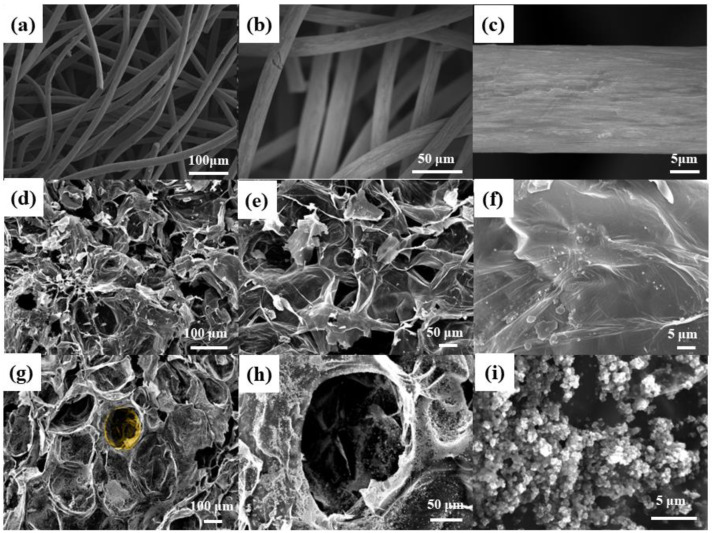
SEM images of blank carbon felt (**a**) 200×, (**b**) 500× and (**c**) 3000×; SEM images of WGCM (**d**) 100×, (**e**) 200× and (**f**) 2000×; SEM images of nano-TiO_2_/WGCM (**g**) 100×, (**h**) 350× and (**i**) 5000×.

The SEM analysis results of the CF, WGCM and nano-TiO_2_/WGCM are shown in Figure 2. From Figure 2a–c we found that the CF was composed of interlaced carbon fibers with clean and smooth surfaces. This network structure could provide more contact sites for the attachment of bacteria, but the smooth surface was not conducive to bacterial adhesion. It could be seen from Figure 2d,e that the layered carbon on the WGCM surface was interconnected to form holes. Freeze drying guaranteed that the porous structure was not destroyed, thereby maintaining the shape structure of macropores and micropores. From Figure 2f it could be concluded that the sheet carbon was continuous with wrinkles on the surface, which increased the surface area. As shown in Figure 2g–i, some particles were uniformly covered on the surface of the WGCM, while some particles were attached on the interior macropores, which increased the roughness of the material. In addition, the sheet carbon on the surface of the nano-TiO_2_/WGCM was reduced, and the micropores were arranged more regularly. It could be inferred that ultrasonic treatment resulted in fracture and detachment of fragile layered carbon because black debris appeared in the solution during this process. SEM indicated that the surface of the modified WGCM was covered with many particles, which made the surface more complex and provided more contact sites for bacteria.

**Figure 3:**
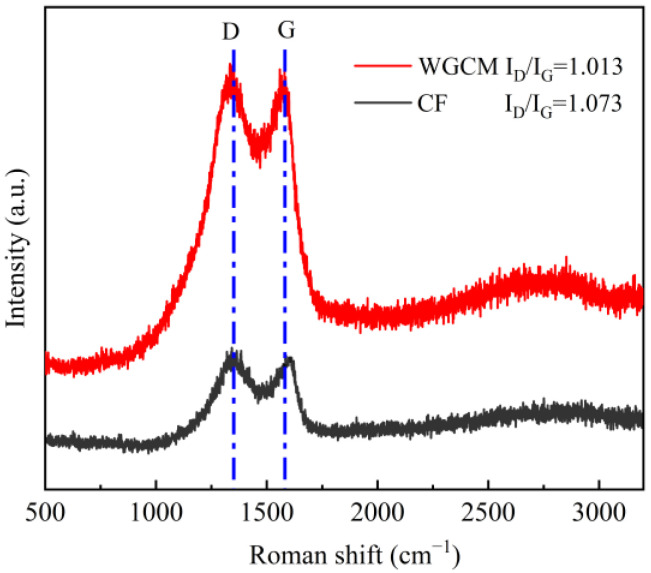
Raman spectra of CF and WGCM.

Raman spectroscopy was used to analyze the crystal structure of the anodes. These were two characteristic peaks in both the CF and WGCM: D peak (defect peak, 1350 cm^−1^) and G peak (graphite peak, 1580 cm^−1^) (Figure 3). Therefore, the ratio of the two peaks (I_D_/I_G_) could be used to evaluate the graphitization degree of carbon materials [25]. The low value of I_D_/I_G_ indicates a high degree of graphitization. The I_D_/I_G_ value of WGCM was 1.013, lower than that of CF (1.073). An enhanced graphitization degree could improve the conductivity of the material [26]. The electrical resistivity of the CF was around 4400 Ωμm, while the WGCM here was greatly reduced to 1080 Ωμm. As a result, the WGCM was expected to enhance the MFC performance due to the improved conductivity.

**Figure 4:**
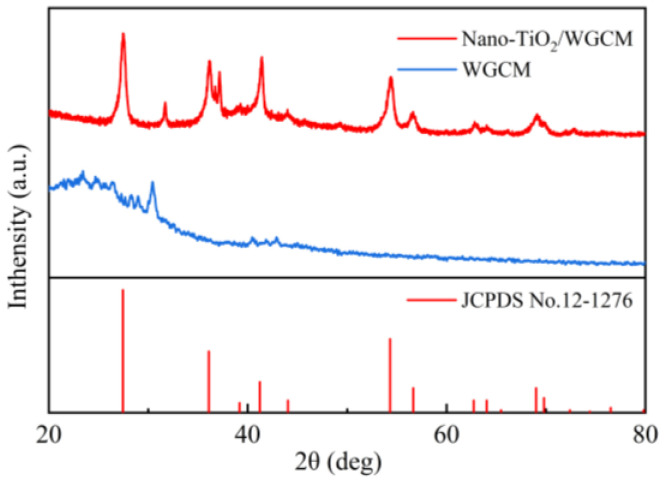
XRD spectrum of WGCM and nano-TiO_2_/WGCM.

The crystal structure of the materials was examined by XRD. In the XRD spectrum of the WGCM (Figure 4), a broad diffraction peak was found at about 26°, corresponding to the (002) crystal planes of the graphite. Nano-TiO_2_/WGCM had strong diffraction peaks at the places where 2 θ = 27.5°, 36.12°, 41.41°, 54.39°, 56.63°, 62.83° and 69.1°, which was in good agreement with the (110), (101), (111), (210), (211), (220) and (301) crystal planes of rutile TiO_2_ (JCPDS, No. 21-1276). These results demonstrated that TiO_2_ was successfully coated on the WGCM.

**Figure 5:**
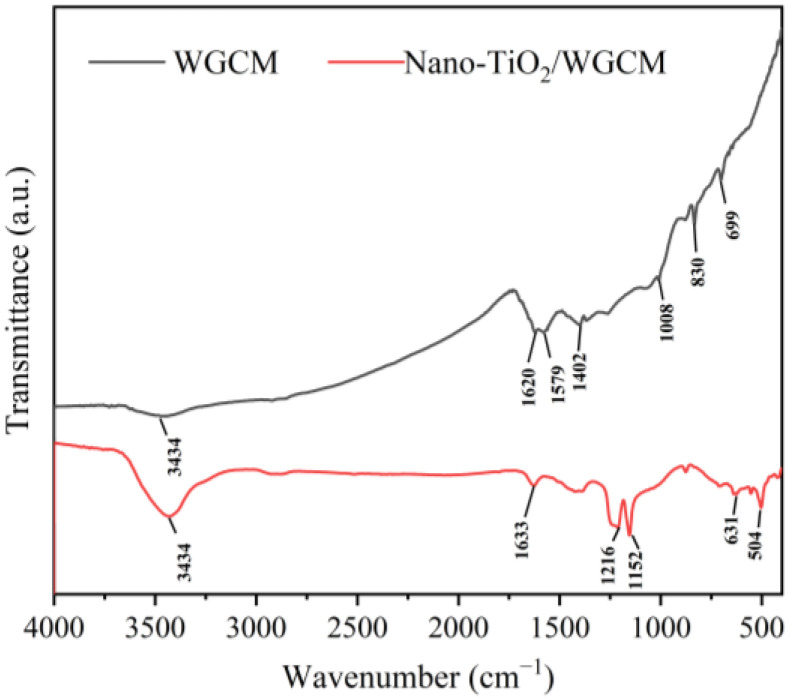
FTIR spectra of WGCM and nano-TiO_2_/WGCM.

FTIR was used to determine the functional groups of the WGCM and the results are shown in Figure 5. In the infrared spectrum of the WGCM, the peak at 3434 cm^−1^ was due to the -OH stretching vibration while the wave number at 1620 cm^−1^ corresponded to the C=O bond stretching vibration. The peak at 1579 cm^−1^ was attributed to the bending vibration of N-H bonds. The peaks at 1402 cm^−1^ and 1008 cm^−1^ were the stretching vibration of C-N bonds and C-O bonds, respectively. In the infrared spectrum of the nano-TiO_2_/WGCM, the -OH stretching vibration peak appeared at 3434 cm^−1^ while the C=O stretching vibration peak was observed at 1633 cm^−1^. The peak at 1216 cm^−1^ was the C-N bond stretching vibration, while the peak at 1152 cm^−1^ was the C-O bond stretching vibration. New distinct peaks appeared at 631 cm^−1^ and 504 cm^−1^, which were attributed to the antisymmetric stretching vibration and symmetric stretching vibration of the Ti-O bond, respectively. These results demonstrated that hydroxyl, carboxylic and amine groups were generated on the WGCM surface. The existence of O-containing or N-containing functional groups could improve the hydrophilicity of the material, which is conducive to the biofilm formation [27]. In addition, N-containing functional groups could boost the electron transfer between the electrode and EAB and improve power generation [28].

**Figure 6:**
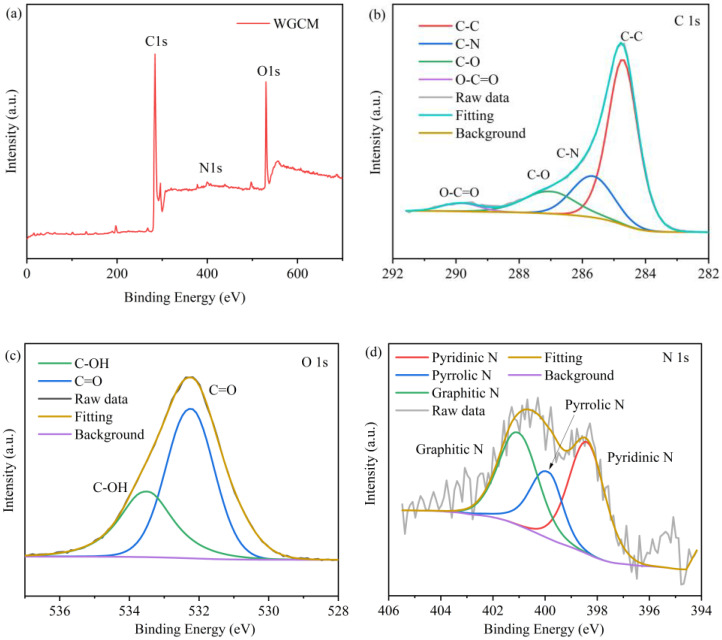
(**a**) Full XPS spectra, (**b**) C 1s spectra, (**c**) O 1s spectra and (**d**) N 1s of WGCM.

The contents of elements and functional groups of the WGCM were determined by XPS, and the results are shown in Figure 6. The parameters of intensity of the C 1s peak (284.1 eV), O 1s peak (530.1 eV) and N 1s peak (399.1 eV) can be used to calculate the elemental composition, which are shown in Figure 6a. The result showing that the contents of C, O and N were 75.5 at%, 22.1 at% and 2.4 at%, respectively. The introduction of nitrogen may be due to the protein content of the wax gourd (0.3 g/100 g), and the carbonization of the wax gourd did not need to be achieved by other methods. Although the N content which played an important role in improving the electrochemical performance of electrode materials was low in the WGCM. The C 1s high-resolution spectrum of the WGCM could be resolved into four peaks (Figure 6b), such as C-C (284.7 eV), C-N (285.7 eV), C-O (286.9 eV) and O-C=O (289.8 eV). The C=O at 532.2 eV and the C-O at 533.5 eV were analyzed by the spectra of O 1S (Figure 6c), which were 63.2 at% and 36.8 at%, respectively. Oxygen-containing groups can improve the hydrophilicity of the material, which was further proved in the hydrophilicity test. Furthermore, the power density of MFCs can be improved by the peptide and hydrogen bonds formed between the amide group of outer membrane c-type cytochromes (OM c-Cyts) and the carboxyl group on the electrode material [7]. Three peaks corresponding to Pyridinic N (398.4 eV), Pyrrolic N (400.0 eV), Graphitic N (401.0 eV) were found by analyzing the spectrum of N 1S, and the contents were 39.7 at%, 24.6 at% and 36.7 at%, respectively. The appearance of these types of nitrogen indicated that N has been successfully doped into the C-ring structure. It is easier for Pyrrolic N and Graphitic N to absorb electrons from OM c-Cyts, and the doping of Pyrrolic N can significantly reduce the thermodynamic and kinetic resistance of DET and MET of EABs [29].

**Figure 7:**
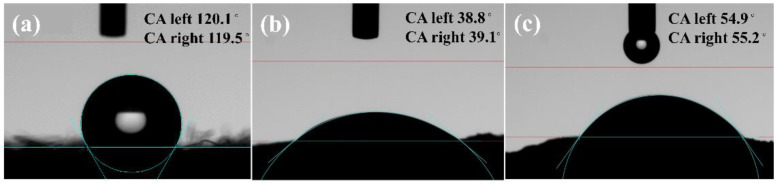
The contact angle of (**a**) CF, (**b**) WGCM and (**c**) nano-TiO_2_/WGCM.

The contact angle evaluated the hydrophilicity of the three electrode materials and the results are shown in Figure 7. The average contact angles of CF, WGCM and nano-TiO_2_/WGCM were 119.8°, 39.0° and 55.1°, respectively. The good hydrophilicity of WGCM and nano-TiO_2_/WGCM was mainly due to the generation of hydroxyl, carboxylic and amine groups on the material surface according to FTIR analysis. The contact angle of nano-TiO_2_/WGCM was slightly larger than that of WGCM, which might be caused by the use of hydrophobic PTFE as an adhesive. Good hydrophilicity is conducive to microbial attachment.

### 3.2. Electrochemical Analysis

**Figure 8:**
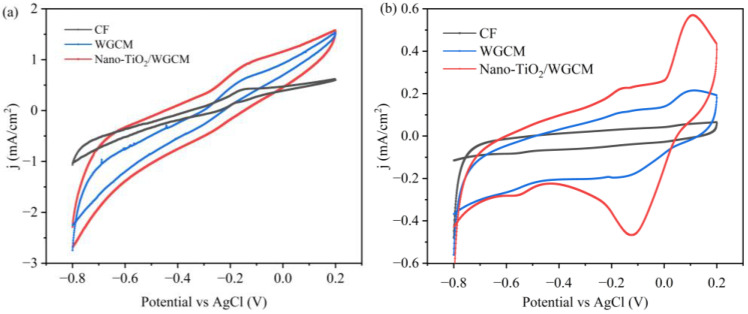
CV of different anodes after biofilm formation (**a**) with acetate and (**b**) without acetate.

The electrochemical behaviors of the CF, WGCM and nano-TiO_2_/WGCM were investigated by CV. Figure 8a shows the CV curves of the bioanode with substrate (acetate); the CV integrated area was positively correlated with electrode capacitance. WGCM increased the capacitance and nano-TiO_2_/WGCM further improved the capacitance, indicating that 3D porous WGCM and nano-TiO_2_ enhanced the capacitive property [30]. The CV curves were all S-shaped, and the oxidation curves augmented dramatically from approximately −0.4 V compared to Ag/AgCl. Specifically, the nano-TiO_2_/WGCM anode generated a peak current density of 0.93 mA/cm^2^ under a scan rate of 50 mV/s, which was 128% and 40% higher than that of CF (0.43 mA/cm^2^) and WGCM (0.70 mA/cm^2^), respectively. The higher the current density (the redox peak), the greater the electrocatalytic activity of the electrogen on the anode surface [31,32]. Therefore, these results indicated that biofilms on the WGCM and nano-TiO_2_/WGCM had a better catalytical activity towards acetate oxidation.

CV curves after substrate depletion were also generated to study the extracellular electron transfer mechanism of the bioanode under a scan rate of 10 mV/s [33] (Figure 8b). The redox peak height had an order of nano-TiO_2_/WGCM > WGCM > CF, which was consistent with the power production in MFCs. The redox peak current indicated that more redox mediators were present in nano-TiO_2_/WGCM MFCs. As to the peak position, an oxidation peak at 0.11 V and reduction peak at −0.13 V were observed in all the MFCs. However, an oxidation peak at −0.18 V and reduction peak at −0.56 V were only shown in nano-TiO_2_/WGCM MFCs. This pair of redox peaks corresponded to OmcZ (OM c-Cyts-OmcZ), indicating that the redox protein (OM c-Cyts) on the cell membrane was in direct contact with the electrode to complete the electron transfer [31].

### 3.3. MFC Performance

**Figure 9:**
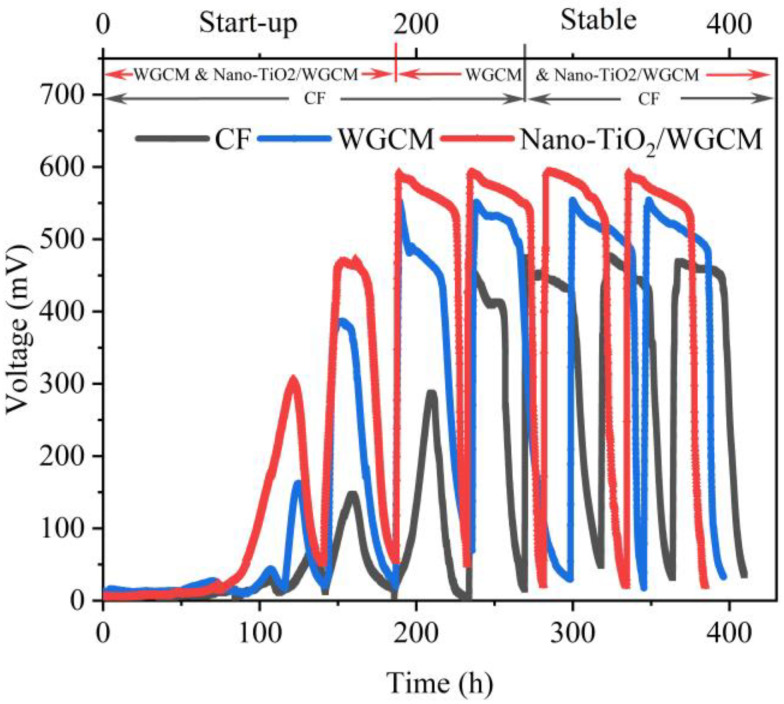
The start-up voltage of MFCs and the stable output voltage of MFCs with different anodes.

**Figure 10:**
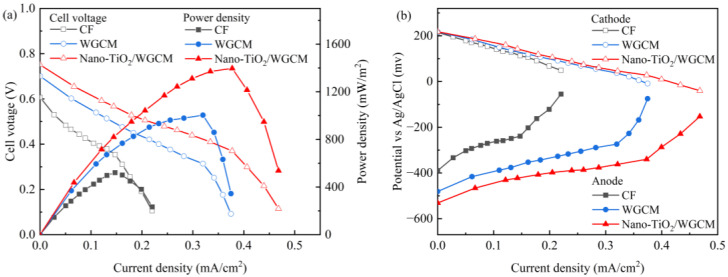
(**a**) The polarization curves and power densities of MFCs based on CF, WGCM and nano-TiO_2_/WGCM; (**b**) electrode polarization curves.

The start-up of the three MFCs are displayed in Figure 9. After eight days (187 h) of inoculation, the output voltage of the WGCM and nano-TiO_2_/WGCM anodes reached a stable state, with a peak voltage of approximately 551 mV and 593 mV, respectively. The start-up time of CF was 11 d (269 h), and the peak voltage reached about 456 mV. The starting times of the WGCM and nano-TiO_2_/WGCM were shorter than that of CF, indicating that the biofilm formed more quickly. In the start-up, the stable voltage was obtained earlier and to a greater magnitude in the nano-TiO_2_/WGCM than in WGCM. In this way, although the nano-TiO_2_/WGCM was slightly less hydrophilic, it was also more conducive to microbial attachment due to the good biocompatibility of TiO_2_ nanoparticles. Moreover, as shown in Figure 9, the stable output voltage of the nano-TiO_2_/WGCM (about 590 mV) was higher than that of WGCM (about 550 mV) and CF (about 450 mV), suggesting that WGCM and nano-TiO_2_/WGCM enhanced the current generation performance of MFCs as they had the same external resistance. As to the power generation and internal resistance, we discuss it in Figure 10.

The power density and polarization curves of MFCs with CF, WGCM and nano-TiO_2_/WGCM anodes are shown in Figure 10a. The results demonstrated that WGCM and nano-TiO_2_/WGCM had obvious impacts on the power generation. The MFCs with an WGCM anode or nano-TiO_2_/WGCM anode had a maximum power density of 957.6 mW/m^2^ and 1396.2 mW/m^2^, respectively, which was much higher than that of the CF MFC (521.0 mW/m^2^). Compared with the CF MFC, the nano-TiO_2_/WGCM MFC and the WGCM MFC enhanced the maximum power density by 167.95% and 45.78%, respectively. Meanwhile, the internal resistance could be calculated from the slope of the polarization curves. The CF MFC, WGCM MFC and nano-TiO_2_/WGCM MFC had an internal resistance of 264 Ω, 219 Ω and 200 Ω, respectively. Figure 10b shows the anode and cathode potential curves of the three MFCs. The three cathodic potential curves were similar, while the anodic potential curves were quite different. Therefore, the difference in power generation of the three MFCs was mainly caused by the anode [34]. These results indicated that WGCM and nano-TiO_2_/WGCM reduced the internal resistance and enhanced the power production of MFCs. The improvement of power density was speculated to be due to nano-TiO_2_/WGCM being more suitable for the attachment of electroactive bacteria. The microbial biomass on the anode surface was then measured.

The performance of the MFCs in this study was compared with that of biomass derived carbon anode MFCs reported previously, as listed in Table 1. The power density of WGCM (bare electrode) in MFCs was 957.6 mW/m^2^, which was good compared with some of the other electrodes in the table. The maximum power density of nano-TiO_2_/WGCM MFC reached 1396.2 mW/m^2^, which was higher than most biomass-derived electrodes listed in the table.

### 3.4. Biofilm on Anodes

**Figure 11:**
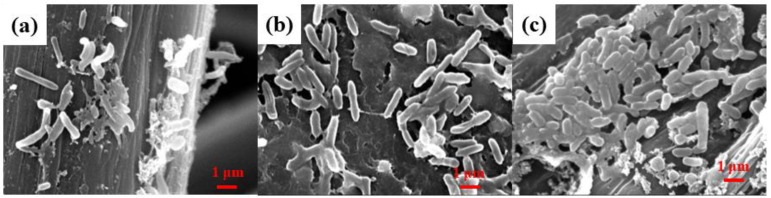
SEM images of biofilms on different anodes (10,000×): (**a**) CF, (**b**) WGCM, (**c**) nano-TiO_2_/WGCM.

SEM was employed to study the biofilm formation on the anode surface. Figure 11 shows that some rod-shaped bacteria were attached on the surface of the CF, while more bacteria were growing on the surface of the WGCM and nano-TiO_2_/WGCM.

The biofilm cell densities on different anodes were further measured by the hemocytometry counting method. The results showed that the cell densities on the CF, WGCM and nano-TiO_2_/WGCM were 4.5 × 10^8^ cells/cm^2^, 7.5 × 10^8^ cells/cm^2^ and 9.4 × 10^8^ cells/cm^2^, respectively, which was in agreement with the SEM study. The results suggested a positive correlation between the maximum power density and the number of bacteria on the electrode surface. The nano-TiO_2_/WGCM exhibited the highest surface bacteria biomass, which produced the highest power density and showed optimal biocompatibility.

**Figure 12:**
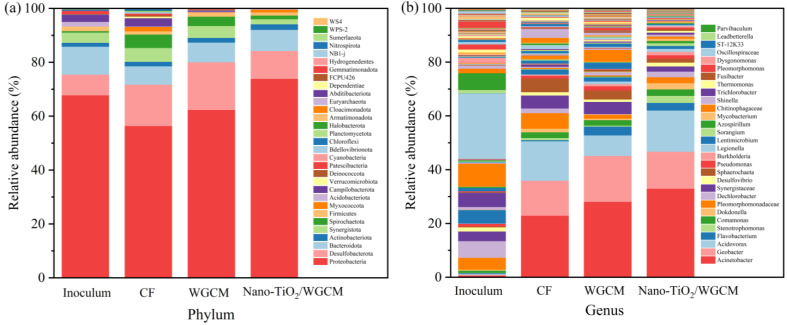
Microbial community composition and abundance of the biofilm at (**a**) phylum and (**b**) genus levels.

Since the electrode surface microorganisms consist of EABs and non-electroactive bacteria, further analysis of the microbial community structure was required. The microbial communities in the different anodic biofilms were analyzed using Illumina Hiseq sequencing of 16S rRNA gene technology (Figure 12a). The dominant phyla in the inoculum were *Proteobacteria* (67.75%), *Bacteroidota* (11.36%) and *Desulfobacterota* (7.65%). *Proteobacteria* (*Acinetobacter*, *Geobacter*, *Pseudomonas*, etc.) and *Desulfobacterota* played important roles in MFCs due to the fact that they were typically the major electricigens in MFCs [40,41]. The abundances of *Proteobacteria* in the CF, WGCM and nano-TiO_2_/WGCM were 56.32%, 62.30% and 78.85%, respectively, indicating that the WGCM and nano-TiO_2_/WGCM were conducive to *Proteobacteria* attachment.

Figure 12b shows the microbial communities of the biofilms at the genus level. *Acinetobacter* was the most abundant bacteria on the three anodes. Specifically, the *Acinetobacter* abundance on the CF was 22.97%, while it was 28.12% on WGCM and 32.99% on nano-TiO_2_/WGCM. *Acinetobacter* is a Gram-negative bacterium, which is widely found in soil, fresh water and other natural environments. It was the dominant genus of MFC anode electrogens [42]. In addition, the abundance of *Geobacter* was also considerably enhanced, it achieved 13.00%, 17.08% and 13.75% on CF, WGCM and nano-TiO_2_/WGCM, respectively. *Geobacter*, one of the most famous genera in MFCs, specialized in coupling organic matter oxidation to the reduction of insoluble iron oxide in soil and sediments and played a significant role in global biogeochemistry. The results demonstrated that WGCM and nano-TiO_2_/WGCM greatly enhanced the microbial cell density of the biofilm on anode surface. Furthermore, WGCM and nano-TiO_2_/WGCM also increased the abundance of *Acinetobacter* and *Geobacter,* two well-known electroactive bacteria in MFCs.

### 3.5. Mechanisms and Novelties of Anode Enhancement

The WGCM anode enhanced the power production in MFCs compared with the CF anode. First, this was due to the 3D microporous network structure as demonstrated by the SEM imaging, which increased the surface area for bacteria adhesion and enabled internal colonization. Second, the hydrophilic surface of the WGCM was also beneficial for biofilm formation. A previous study showed that enhanced hydrophilicity would facilitate the electroactive biofilm formation and consequently improve the power production in MFCs [43]. Here, the contact angle test demonstrated that the WGCM had a much higher hydrophilic surface than CF, owing to the hydroxyl, carboxylic and amine groups generated during carbonization. The WGCM was rich in oxygen-containing and nitrogen-containing functional groups. Due to these two reasons, the cell density was greatly increased from 4.5 × 10^8^ cells/cm^2^ on CF to 7.5 × 10^8^ cells/cm^2^ on WGCM. Lastly, the high conductivity of WGCM was advantageous to the anode enhancement. The I_D_/I_G_ value of the WGCM (1.013) was lower than that of CF (1.073), suggesting a higher degree of graphitization [44,45]. The electrical resistivity of the WGCM (1080 Ωμm) was much smaller than that of the CF (4400 Ωμm). The enhanced conductivity benefited the anodic electron transfer and reduced the internal resistance as demonstrated in Figure 10.

TiO_2_ further enhanced the anode performance in MFCs. This was probably due to the enhanced enrichment of *Acinetobacter* on the nano-TiO_2_/WGCM. In this study, the microbial cell density increased from 7.5 × 10^8^ cells/cm^2^ on WGCM to 9.4 × 10^8^ cells/cm^2^ on nano-TiO_2_/WGCM, and the abundance of *Acinetobacter* also grew from 28.12% on WGCM to 32.99% on nano-TiO_2_/WGCM. Calculated from the cell density and the abundance, TiO_2_ increased *Acinetobacter* in the biofilm by 47.0%. *Acinetobacter* species are well-known exoelectrogens in MFCs [46]. For instance, *Acinetobacter* was dominant in the anode microbial community of MFCs constructed with fruit waste residue as the substrate [47] and anaerobic fluidized bed MFCs constructed with industrial wastewater [48]. The study on the degradation of dye wastewater by MFCs showed that the electricity production of MFCs inoculated with *Acinetobacter pitii* was even better than that of MFCs inoculated with mixed bacteria [42].

**Figure 13:**
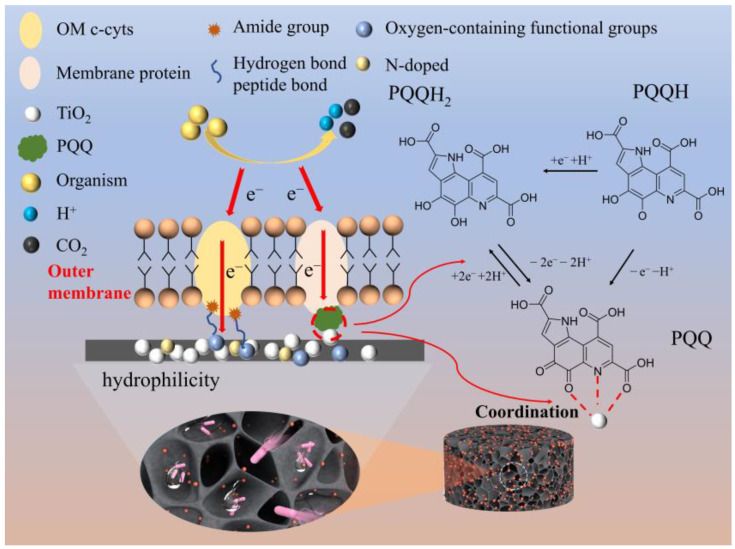
The mechanisms for nano-TiO_2_/WGCM enhancement.

*Acinetobacter* contain pyrroloquinoline quinone (PQQ)-dehydrogenase complexes located in the periplasm of their cells. PQQ was characterized as a redox cofactor for membrane-bound dehydrogenases. The complex is part of the respiratory chain which is responsible for the degradation of organic matter. Karthikeyan et al. constructed MFCs with *Acetobacter aceti* and a nickel anode and demonstrated the electron transfer from the bacteria to the anode via the PQQ-dehydrogenases complex [49]. PQQ, acting as a mediator, could be reduced to its quinol form of PQQH_2_ by accepting two electrons and can be oxidized by releasing two electrons to the anode surface. In Figure 8b, all the CVs showed a redox peak with a midpoint potential of 0 V (Epa = 0.11 V and Epc = −0.11 V), which matched well with the previously reported values of the PQQ-dehydrogenases complex in MFCs [49]. Therefore, the high amount of *Acinetobacter* on the nano-TiO_2_/WGCM enhanced the anode performance. Furthermore, PQQ could provide several binding sites for metal cations, and the existence of coordination of unsaturated Ti (IV) atoms on the surface of TiO_2_ particles could provide a center for PQQ to attach to and consequently promote the EET from PQQ to TiO_2_ (Figure 13) [50]. The strong interaction between the redox center of the membrane protein of the EAB and the electrode was enhanced by TiO_2_ nanoparticles (as a medium), which showed a higher redox peak in Figure 8b. Therefore, the improvement of EET could be attributed to the synergistic effects of the 3D porous WGCM structure and TiO_2_ nanoparticles.

## 4. Conclusions

A 3D porous carbon monolithic electrode was prepared from a wax gourd by freeze-drying and carbonation and then coated with nano-TiO_2_. WGCM and nano-TiO_2_/WGCM microporous structures were beneficial for EAB attachment and quick start-up. In addition, the output voltage and maximum power density of MFCs using WGCM and nano-TiO_2_/WGCM anodes were significantly enhanced compared with the CF control group, profiting from the 3D structure of WGCM, good hydrophilic surfaces and high conductivity. The excellent performance of the nano-TiO_2_/WGCM was due to the enrichment of the electrogen *Acinetobacter*, which further improved the output power. In summary, the 3D monolithic carbon material coated with nano-TiO_2_ is a promising anode material.

## Figures and Tables

**Table 1:** Comparison of the performance of MFCs with different biomass-derived carbon.

Anode Materials	Surface Area of Electrodes (cm^2^)	Size of Electrodes	Inoculum	Power Density (mW/m^2^)	References
KOH-activated chestnut shells	125.65	0.3 × 66.4 cm	Anaerobic mixed sludge	850	[35]
N–P-doped onion peels	7	1.0 × 2.0 × 0.5 cm	Mix sludge	742	[36]
Basswood	4	1.0 × 2.0 × 0.1 cm	Shewanella oneidensis MR-1	483	[37]
PEDOT/NiFe_2_O_4_/ Neem wood	1	1 × 1 cm	Pre-acclimated wastewater	1200	[38]
PANI/ Steamed cake	18.70	7 cm^2^ × 0.5 cm	Effluent from another stable operation of MFCs	1307	[39]
WGCM	12.17	Φ 2.5 × 0.3 cm	Effluent from active acetate-fed MFCs	957.6	This study
Nano-TiO_2_/WGCM	12.17	Φ 2.5 × 0.3 cm	Same as above	1396.0	This study

## Data Availability

Not applicable.
